# Shear‐Convection Interactions and Orientation of Tropical Squall Lines

**DOI:** 10.1029/2021GL095184

**Published:** 2021-12-29

**Authors:** Sophie Abramian, Caroline Muller, Camille Risi

**Affiliations:** ^1^ Laboratoire de Météorologie Dynamique IPSL CNRS Ecole Normale Supérieure Sorbonne Université PSL Research University Paris France; ^2^ Now at Institute of Science and Technology Austria (IST Austria) Klosterneuburg Austria

**Keywords:** squall lines, radiative convective equilibrium, deep convection, cold pools, wind shear

## Abstract

Squall lines are known to be the consequence of the interaction of low‐level shear with cold pools associated with convective downdrafts. Also, as the magnitude of the shear increases beyond a critical shear, squall lines tend to orient themselves. The existing literature suggests that this orientation reduces incoming wind shear to the squall line, and maintains equilibrium between wind shear and cold pool spreading. Although this theory is widely accepted, very few quantitative studies have been conducted on supercritical regime especially. Here, we test this hypothesis with tropical squall lines obtained by imposing a vertical wind shear in cloud resolving simulations in radiative convective equilibrium. In the sub‐critical regime, squall lines are perpendicular to the shear. In the super‐critical regime, their orientation maintain the equilibrium, supporting existing theories. We also find that as shear increases, cold pools become more intense. However, this intensification has little impact on squall line orientation.

## Key Points

- In the subcritical regime, squall lines are perpendicular to the wind shear, until equilibrium between wind shear and cold pool spreading
- In the supercritical regime, the orientation of the squall lines reduces the incoming wind shear, and maintains the equilibrium
- Changing properties of cold pools with background shear, notably their deepening, has little effect on the squall line orientation

## 1 Introduction

Squall lines are bands of thunderstorms of hundreds of kilometers, also called quasi‐linear mesoscale convective systems. One key ingredient in the organization of squall lines is the presence of cold pools below precipitating clouds. These are areas of cold air with negative buoyancy anomaly, driven by the partial evaporation of rain and concomitant latent cooling, and observed to span 10–200 km in diameter (Romps & Jeevanjee, 2016; Zuidema et al., 2017). Cold pools spread radially at the surface as gravity currents, and can thus favor upward motion and the development of new deep convective cells at their edge as described in Tompkins (2001a) and impact aggregation (Muller & Bony, 2015).

**Figure 1:**
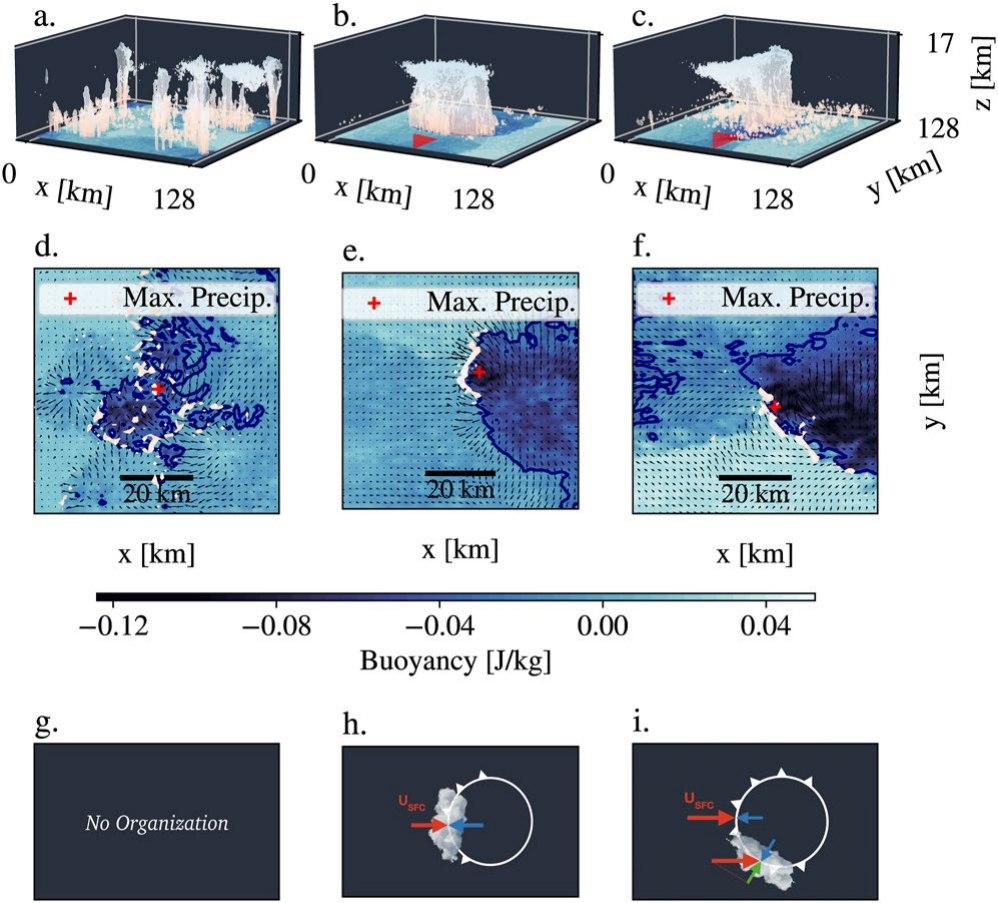
(a, b, c) 3D overview of the control case *U*_sfc_ = 2.5 m s^−1^, near critical case *U*_sfc_ = 10 m s^−1^ and supercritical case, *U*_sfc_ = 17.5 m s^−1^. Buoyancy is displayed on the ground and the isosurface of cloud humidity equal to 0.02 mm is in white. (d, e, f) Top view of the three cases described above, zooming around the location of maximum precipitation (showing subregions with 70 km side). The colormap is the buoyancy, the quiver field represents the velocity field and the white area corresponds to the positive vertical velocity at 300 m. (g, h, i) Theoretical representation. For the control case, no organization is expected. In the critical case, the cold pool frontaly counterbalances the shear. In the supercritical case, the line tends to orient itself to conserve the projected wind (green).

Based on observations (Bluestein & Jain, 1985; Chalon et al., 1988; Chong et al., 1987; Houze, 1977; Zipser, 1977), a theory for squall lines was constructed by Rotunno et al. (1988) (hereafter RKW; see also Garner and Thorpe, 1992; Weisman and Rotunno, 2004), which is still the standard for their development today. RKW starts from the fact that in the absence of wind shear, deep convection developing at the edge of cold pools has a tilted updraft, and hence can not easily develop (see RKW notably their Figure 18; Figures 1a, 1d, and 1g).

One key parameter for the organization of deep convection into squall lines, is therefore the strength of cold pools and associated density currents, compared to the strength of the background wind shear. Depending on the shear amplitude, and assuming that the properties of the cold pools do not change with the shear, one can thus expect three regimes: the sub‐critical regime, where density currents dominate, the critical regime where equilibrium is reached, and finally the super‐critical regime where shear dominates. In the latter case, the squall lines tend to orient themselves at an angle to the shear (Figures 1c, 1f, and 1i). The literature suggests that the orientation of the line keeps the projected component of the shear close to the critical value, a hypothesis that we further investigate here.

Robe and Emanuel (2001) investigated the evolution of the squall line organization for a range of shears. In this numerical study and also in observational cases (Chong, 2010; Coniglio et al., 2012; LeMone et al., 1998), RKW theory is widely accepted. However, the various models and observations are mainly confined to subcritical or critical speeds, and lack of quantitative and objective measure of angles. Indeed, it is difficult to assess quantitatively the organization of the lines, since they often form cloudy arcs, defining an orientation remains an obstacle. Another aspect that challenges the theory of RKW, suggested this time by Alfaro (2017), is that the strongest squall lines may produce the most intense cold pools and can thus shift the optimality regime and also modify the orientation of the lines.

The objective of our study is to clarify the physical processes responsible for the organization of deep clouds into squall lines in the tropics, and quantitatively test the RKW hypothesis up to the supercritical regime, in order to determine whether the cold pool and shear balance is sufficient to describe squall lines in cloud‐resolving simulations. More precisely:

1. How to measure the orientation of the squall lines ?
2. Does the orientation of the lines match the RKW hypothesis?
3. Is it wrong to consider that the cold pools properties do not change with the shear?

The next section describes the cloud‐resolving simulations, and the method to detect the angle of squall lines with respect to the background wind shear. Section 3 describes expectations from the RKW theory in more details, and investigates its valadity. Section 4 investigates the sensitivity of cold pools to shear strength, and its impact of squall line orientation. Concluding remarks are offered in Section 5.

## 2 How to Measure the Orientation of the Squall Lines?

Before addressing the RKW theory in detail, the objective of this section is to describe the methodology that leads us to the estimate of the squall line angle with respect to the shear. Robe and Emanuel (2001) attempted such an angle detection, but using visual inspection. Here instead, we will derive a reproducible method based on image autocorrelation. We recall the set‐up of the simulations (see Supporting Information S1 for additional details) and then present our angle detection algorithm.

### 2.1 Model and Simulations

The simulations use the cloud‐resolving model SAM (Khairoutdinov & Randall, 2003). The resolution is 1 km in both horizontal directions, and gradually increasing in the vertical direction from 80 m near the surface to 400 m above 6 km. The 3D domain is doubly periodic in *x* and *y* with 128 km side, and the upper third of the domain (18–27 km) has a sponge layer to absorb gravity waves. We neglect the Earth rotation (a reasonable approximation in the tropics). All simulations are run to radiative‐convective equilibrium (reached in about 30 days), after which we start our analysis, from day 30–35, with hourly outputs.

Following Muller (2013), the convection is organized into squall lines by imposing a background vertical wind shear profile in the *x*‐direction, with a background wind decreasing linearly from *U*_sfc_ at the surface to 0 m s^−1^ at *z* = 1 km. We perform nine simulations, with different shear strengths, that is, with different surface wind *U*_sfc_ from 0 (no shear) to 20 m s^−1^, with 2.5 m s^−1^ increments. To avoid an impact of this imposed surface wind on surface fluxes, the domain‐mean surface wind is removed before computing surface fluxes (see Supporting Information S1 for more details on the simulations).

Figure 1 shows three of our simulations. The 3D top graphs show buoyancy on the ground, and convective clouds in white. The 2D middle graphs show the same snapshots but from a top view and zoomed near the maximum precipitation. The bottom panels show a schematic of cold pool spreading (curve with triangles) and deep convection triggered at its edge, with increasing low level shear (*U*_sfc_ = 10 m s^−1^ middle panel, and 17.5 m s^−1^ right panels). With shear, convection triggering is favored in the downshear (decreasing *x*) direction, as described in more detail in Section 3.

### 2.2 Automatic Measurements of Squall Lines Orientation

**Figure 2:**
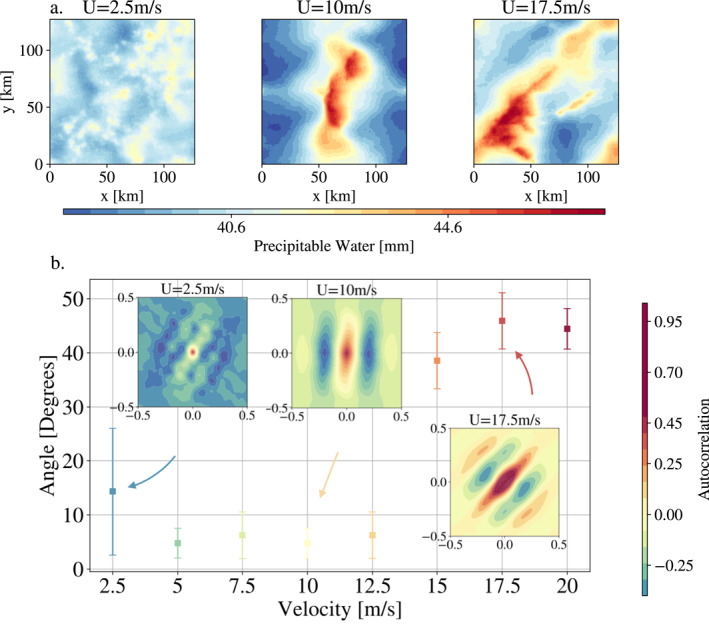
(a) Snapshots (31st day) of the spatial distribution of Precipitable Water (PW) for different shear values. In the absence of shear, no squall lines are observed. For *U*_sfc_ = 10 m s^−1^, arcs perpendicular to the wind, that is, parallel to the *y*‐axis, are formed. For *U*_sfc_ = 17.5 m s^−1^, the squall line is oriented with respect to the *y*‐axis. (b) Results of the automatic method based on PW autocorrelation developed in this paper to detect the angle *a* between the squall line and the *y*‐axis. The auto‐correlations of PW corresponding to the three top snapshots are also displayed; they are normalized and the colorbar is saturated to highlight the correlation areas.

We define the angle *a* of the squall line orientation, as the angle between the squall line and the *y* direction. This definition allows to have a reference angle of 0 when the line is perpendicular to the background wind. To measure this angle *a*, we investigate the spatial autocorrelation of precipitable water (PW, which is the vertically integrated water vapor amount, shown Figure 2 top panels). We choose this variable because PW has a long time persistence compared to other variables (for instance vertical velocity, or precipitation). Thus it has a longer memory of convective events and allows the mesoscale line organization to clearly appear.

Figure 2 provides a summary of the automatically estimated angle for each case (between 0 and *π*/2; see Supporting Information S1 for more details on the angle computation, notably Figure S1 in Supporting Information S1 for time series of the squall line angle). Three PW snapshots are displayed on top, with the corresponding autocorrelation images below. Figure 2 highlights (a) The absence of a precise angle for the control case; (b) Two regimes of the squall lines, the sub‐critical one, where the lines are perpendicular to the wind (an angle close to 0) and the super‐critical regime where the lines are oriented from 40 to 45°; (c) A critical case for *U*_sfc_ = 12.5 m s^−1^, the last case before the orientation of the line.

We now compare these estimated angles to the theory of squall line orientation.

## 3 Does the Orientation of the Squall Lines Match the RKW Hypothesis?

In this section, we use our simulations to understand the physical mechanisms behind the formation of tropical squall lines, and their orientation. The framework of RKW and Robe and Emanuel (2001) provides a theoretical approach of this phenomenon, however the agreement of the orientation with this theory has never been clearly demonstrated. Using our angle measurement from Section 2, we quantitatively test RKW theory.

### 3.1 RKW Theory and Orientation of the Squall Lines

Figure 1a (control case) shows that without shear, convection develops randomly over the domain, similar to popcorn formation. In the presence of shear, for instance in the case *U*_sfc_ = 10 m s^−1^ (panels b, e, h) we can see a deep convective line, with a length of 75 km, up to 10 km of altitude. The shear seems to constrain the convective cells at the edge of the cold pool. This is consistent with the aforementioned RKW theory of equilibrium between the incoming wind shear and that associated with spreading cold pools.

In the case *U*_sfc_ = 10 m s^−1^, the line is perpendicular to the imposed wind, suggesting that it is a critical case (see also Figure 2). This means that the cold pool propagation is of the same order as the imposed background wind. In this case the location of the convective cell triggering, is on the extreme downshear front of the pool.

When the surface wind becomes stronger, we move into the super‐critical regime. Considering the panel *U*_sfc_ = 17.5 m s^−1^, we observe a deep convective line, oriented with respect to the imposed wind.

Consistent with RKW and Robe and Emanuel (2001), the convection still takes place at the edge of the cold pools, however, the optimal position is not at the downshear front anymore, because the wind is too strong compared to the cold pool propagation. The equilibrium position moves along the edge of the cold pools, such that cold pool velocity remains the same, but the wind projected radially becomes weaker (Figure 1i). In other words, the position of the equilibrium corresponds to the conservation of the projected wind near the cold pool propagation value; the critical one. Note that the equilibrium position is indifferently to the right (increasing *y* direction) or to the left (decreasing *y* direction) of the cold pool, which sometimes gives rise to a V‐shaped pattern, with two lines, one to the left and one to the right of the cold pool.

In short, in the presence of shear, the triggering position of the convective cells is always at the downshear edge of the cold pool, and as the shear rate increases, so does the angle to the *y*‐axis. This orientation is believed to keep the projection of the background wind perpendicular to the squall line direction, close to the propagation speed of cold pools. In the following, we propose to quantify this with the angle detection described in Section 2.

### 3.2 Validation of RKW Theory for Squall Lines Orientation

**Figure 3:**
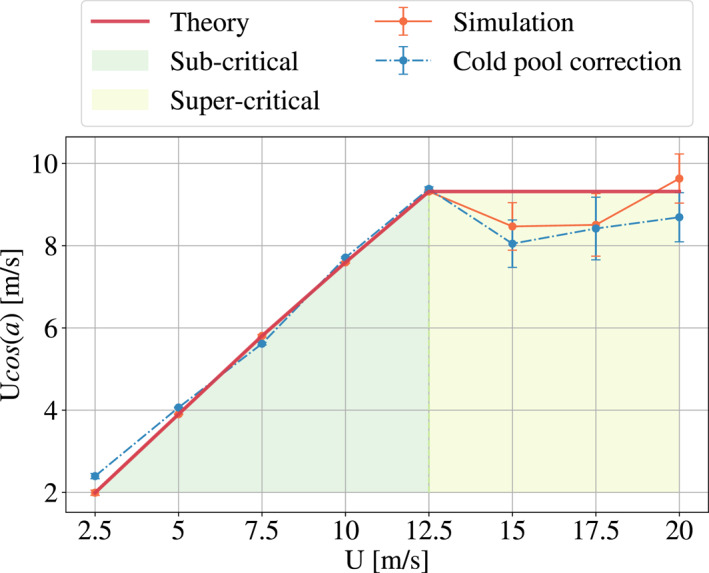
Evolution of the shear basal velocity projection perpendicular to the squall line, according to the eight simulated cases. A very good agreement between the RKW theory and Robe and Emanuel (2001) (red) and our results (orange) is found. We observe a saturation level, separating the subcritical and supercritical shears. The critical state is found for *U*_sfc_ = 12.5 m s^−1^. In our estimates of *U* cos(*a*), we used the actual simulated horizontal mean wind speed in the *x* direction at the first atmospheric level (which is slightly lower than the imposed target velocity toward which the horizontal mean surface wind is relaxed). The dashed blue line shows the same projection when the intensification of cold pools with shear is accounted for.

Based on the angles estimated from the different simulations, several conclusions of RKW theory have already been validated: the existence of two regimes, subcritical and supercritical, and the emergence of a critical shear near 12.5 m s^−1^. To quantitatively test the theory of squall line orientation, we plot the projection of the basal shear velocity perpendicular to the squall line *U*_sfc_  cos(*a*) in Figure 3 for the different simulation cases. The basal velocity is the average over the entire domain of the longitudinal component of the surface velocity. The angle that the wind speed makes with the direction perpendicular to the line corresponds to the angle determined by our method (Figure 1 bottom right panel).

On Figure 3, we also show what is predicted by the theory (in red). For the sub‐critical regime, we should have an increasing line with slope 1, which corresponds to a zero angle; the lines are perpendicular to the wind in order to maximize the incoming wind. This line reaches the critical case, for *U*_sfc_ = 12.5 m s^−1^, and at this moment, the wind speed and the cold pool spreading speed are equal. We then enter the super‐critical regime, and we expect saturation, that is, the angle of the line maintains the cold pool/background wind equilibrium. In orange, we have plotted the results of our simulations. We observe a very good agreement in the sub‐critical regime. In the supercritical regime, we also have a reasonable agreement with the theory, roughly within error bars. At this stage, we can conclude that the theory of the conservation of the shear projection on the direction perpendicular to the squall line allows to predict the orientation angle of the convective bands. We note in passing that the results are robust to reasonable changes in the relaxation timescale used to impose wind shear and to a prescribed large‐scale upward vertical velocity, following (Risi et al., 2021).

However, there is some discrepancy between the theory and our results in the supercritical regime, and we wanted to understand if this was really due to measurement uncertainty or if some other phenomenon was at stake. In particular, it was hypothesized that the potential change of cold pool properties with background shear could feed back on the squall line orientation (Alfaro, 2017). Thus in the next section, we investigate in more detail the cold pools in our simulations.

## 4 Is It Wrong to Consider That the Cold Pools Properties Do Not Change With the Shear?

Cold pools have received increasing attention in observational studies (Feng et al., 2015; Touzé‐Peiffer et al., 2021), and in climate modeling (Hourdin et al., 2020) due to their contribution to triggering new convection. In this section, we describe how cold pools properties evolve as the shear increases, and discuss its consequences on the cold pool‐shear equilibrium that forms squall lines.

### 4.1 Cold Pools Intensification

**Figure 4:**
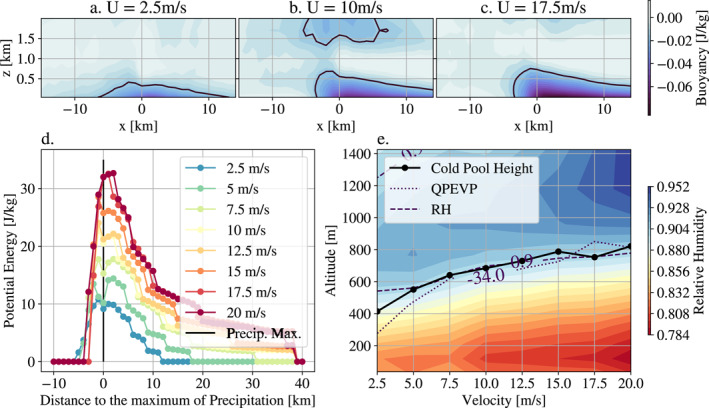
(a, b, c) Composite images of the buoyancy in (*x*, *z*) cross sections around the location of maximum precipitation (located at *x* = 0 in those composites). The compositing is performed at maximum precipitation, over 5 days of radiative‐convective equilibrium with hourly outputs. In black, the boundary *b* = −0.02 K is drawn and delimits the cold pool. (d) Potential energy of cold pools for each shear as a function of the distance to the maximum precipitation. The potential energy is computed at each *x*‐location as the integral of the buoyancy up to the top of the composite cold pool. (e) Cold pool height (estimated as the highest cold pool upper boundary on the composites) as a function of shear, as well as contours of relative humidity RH = 0.9 and rain evaporation QPEVP = −34 g kg^−1^ day^−1^ (estimated as averages within 10 km of the maximum precipitation location).

To analyze the properties of cold pools, and how these may change with shear, we investigate buoyancy anomalies in the vicinity of maximum precipitation at each time step, and then average in time. These composites are shown in Figure 4a. for different shear cases.

With low shear (left), we observe a cold pool nearly symmetrical to the maximum precipitation and not very intense. In the case *U*_sfc_ = 10 m s^−1^, we observe a dissymmetry of the pool, which reflects a resistance of the pool to the frontal wind. Finally, in the super critical case *U*_sfc_ = 17.5 m s^−1^, we observe the same asymmetry, noting that the cold pool seems colder, and deeper.

To quantify this from our composite figures, we compute the cold pool potential energy in J kg^−1^ (following Rotunno et al., 1988; Meyer and Haerter, 2020), defined as

(1) $$E_{p}=\int_{cp}B\left(x,z\right)dxdz$$

where *cp* denotes the domain inside the cold pool, and *B*(*x*, *z*) the composite value of buoyancy in (x,z) cross section around the location of maximum precipitation (see Figure 4a, 4b, and 4c). We define the cold pool upper boundary as the *b* = −0.02 K buoyancy contour (following Tompkins, 2001b), black contour Figure 4 top panels), and integrate (Equation 1) from the surface to this upper boundary.

Figure 4b shows the potential energy of the cold pools for each simulation, as a function of the distance to the maximum precipitation. At the precipitation maximum, a gradual increase in potential energy is observed as a function of the imposed shear. This figure shows an intensification of cold pools.

How to explain this intensification? The intensification is due to the rain evaporation intensification with shear (see Figure S3 in Supporting Information S1), which provokes both an increase in cold pool buoyancy anomaly and in the cold pool height. However, the latter is found to dominate (Figure S2 in Supporting Information S1): the latent cooling is more intense and so downdrafts reach the isoline *b* = −0.02 *K* (cold pools upper boundary) higher in altitude. To determine why rain evaporation increases with shear, we show the evolution of relative humidity vertical profiles (estimated as averages within 10 km of the maximum precipitation location) for all shear cases (Figure 4). We observe that the top of the dry layer deepens monotonically with the shear (dashed line), and this trend is highly correlated with the rain evaporation (dotted line) and eventually the cold pool height (solid line). We found that 60*%* of the increase in rain evaporation between the highest shear case and the control one is due to a drier environment (see Text S4 in Supporting Information S1). We hypothesize that it can be explained by the meso‐scale circulation which affects the relative humidity profiles.

### 4.2 Impact on Squall Lines Orientation

Does this intensification of cold pools impact the squall line orientation? The intensification of cold pools, insofar as they are associated with a higher propagation speed, could move the position of triggered convective cells downshear. More precisely, if we make the hypothesis of a total transfer of potential energy to horizontal kinetic energy of cold pool spreading, we can deduce a propagation speed of the pools, (Benjamin, 1968; Meyer & Haerter, 2020; Rotunno et al., 1988)

(2) $$v_{p}=\sqrt{2E_{p}}.$$

Since the potential energy increases with the imposed shear (see Figure 4), so does the velocity. We can take into account this increase of velocity in the equilibrium between cold pools and background shear, in order to bring a correction to the current theory. More precisely, we account for the cold pool velocity deviation from the critical case, that is, the correction brought by the cold pools is $v_{p}^{\prime }=v_{p}-v_{p}^{crit.}$. This lead to the following equilibrium,

(3) $$U_{sfc}\cos\left(a\right)∼U_{sfc}^{crit.}+v_{p}^{\prime }$$

where $U_{sfc}^{crit.}$ corresponds to the critical case 12.5 m s^−1^ Figure 3 shows in blue the correction brought by this calculation $U_{sfc}\cos\left(a\right)-v_{p}^{\prime }$ (instead of *U*_sfc_  cos(*a*)). For the subcritical regime, we still have the same agreement. For the supercritical case, we observe that the correction brings the theoretical expectation in closer agreement with the CRM simulations. Since this correction is small, we conclude that the intensification of the cold pools is not a determining factor for the orientation of squall lines.

## 5 Conclusion

While the RKW angle theory was so far qualitatively supported, for the first time we support it with an objective and reproducible quantification of squall line orientation. Using autocorrelation image analysis of the integrated humidity profiles, we measure the orientation from CRM simulation of the squall lines and identify the subcritical and supercritical regimes, depending on the ratio between the basal shear velocity, and the spreading velocity of cold pools. The hypothesis of the conservation of the projected basal wind perpendicular to the squall line, that predicts the orientation of squall lines, is here validated.

We further investigate the properties of cold pools, and notably their evolution with shear. We measure an intensification and a deepening of the cold pools due to the drying of the lower atmosphere with increasing shear. However, the resulting intensification of the cold pools has a secondary impact on the orientation of the line.

Although these results are within an idealized framework, they draw our attention to the sensitivity of convective organization to wind shear. Squall lines are associated with extreme rainfall, and an interesting question could be the impact of squall line orientation on extreme precipitation rates. In the longer term, more work is desirable to extend these results beyond the CRM framework, for example, by using satellite data (Fiolleau & Roca, 2013; Roca & Fiolleau, 2020). The deployment of satellite observations at fine‐scale resolution (kilometers, for example within cold pools), such as those proposed in the ESA Harmony mission (the only Earth Explorer 10 candidate mission), would be essential to perfect our understanding of the processes (Harmony, 2020).

## Supporting information

Supporting Information S1

Movie S1

## Data Availability

The simulation data that support the findings are available in Figshare (https://doi.org/10.6084/m9.figshare.c.5705909.v1). The different algorithms and the main script developed in this article are freely available on the Zenodo SAM_project repository (https://doi.org/10.5281/zenodo.5078167), including the pySAM Python package.
