# Supplementary material for: Shear‐Convection Interactions and Orientation of Tropical Squall Lines
Source: Geophys Res Lett. 2021 Dec 29;49(1):e2021GL095184. doi: 10.1029/2021GL095184 (PMC9287078; doi:10.1029/2021GL095184)
Supplement: Supplementary file 1 — Supporting Information S1 [file GRL-49-0-s001.pdf]

Supporting Information for "Shear-convection interactions, and orientation of tropical squall lines"

Sophie Abramian[1], Caroline Muller[1], Camille Risi[1]

[1] Laboratoire de Météorologie Dynamique, IPSL, CNRS, Ecole Normale Supérieure, Sorbonne Université, PSL Research University, Paris, France

Contents of this file

1. **Model and simulations details.** In this section, we provide more detailed information on the cloud-resolving model used and the specific configurations of the simulations.

2. **Automatic detection of squall line angle: method description.** In this section, we describe in detail the method used in the paper to automatically detect the angle orientation of the squall line.

3. **Investigation of cold pool intensification with increasing background shear.** In this section, we analyze in more detail the changes of cold pool properties as the background wind is increased, and split this change into a change of cold pool depth and a change of cold pool averaged buoyancy anomaly.

4. Investigation in cold pools, what are the origins of their intensification?

In this section, we describe the average relative humidity and rain evaporation profiles above cold pools. This can help understanding the link between the cold pool height and the rain evaporation altitude.

1. Model and simulations details

We simulate several cases of squall lines using the Cloud-Resolving Model (CRM) System for Atmospheric Modeling, or SAM (?, ?). This model is based on a non-hydrostatic and anelastic formulation of atmospheric flows. It has the ability to cover a wide range of scales, from deep convective kilometric scale to mesoscale organization $\mathcal{O}$(100s km), and is thus adapted to the study of mesoscale systems such as squall lines. The prognostic thermodynamic variables of the model include total nonprecipitating water (vapor, cloud water, cloud ice) and total precipitating water (rain, snow, graupel). The mixing ratios of cloud water, cloud ice, rain, graupel, and snow is diagnosed from the prognostic variables using a temperature-dependent partition between liquid and ice phases. The frozen moist static energy, which is the sum of the liquid/ice water static energy and the total condensate amount times the latent heat of vaporization, is conserved during moist adiabatic processes in the model, including the freezing and melting of precipitation. The model is run to radiative-convective equilibrium, and once equilibrium is reached (in about 30 days) the organization of squall lines is analyzed, from day 30 to 35 with hourly outputs.

All simulations are three-dimensional on a square, doubly periodic horizontal domain, with horizontal resolution 1 km and domain size 128 km in x and y directions. The vertical grid has 64 levels (capped at 27 km with a rigid lid), with the first level at 37.5 m and grid spacing gradually increasing from 80 m near the surface to 400 m above 6 km, and a variable time step (10 s or less to satisfy the Courant–Friedrichs–Lewy condition). The surface fluxes are computed using Monin–Obukhov similarity. To reduce gravity wave reflection and buildup, a sponge layer with Newtonian damping is applied to all prognostic

variables in the upper third of the model domain, from 18 to 27 km. We neglect the Earth rotation, a reasonable assumption in the deep tropics where the Coriolis parameter is small (set to zero in our simulations).

To organize the deep convection into squall lines, a linear shear in the x direction is imposed (following ? (?)). The imposed profile has a wind in the x-direction decreasing from U_{sfc} at the surface $z = 0$ km to $U_{1km} = 0$ m s^{-1} at $z = 1$ km. This background shear is imposed by relaxing the mean wind towards this profile with a relatively fast time scale of 2 hours. Nine simulations, with the surface wind U_{sfc} varied from 0 (no shear) to 20 m s^{-1}, with 2.5 m s^{-1} increments, are performed. To avoid an impact of this imposed surface wind on surface latent and sensible fluxes (which are proportional to surface wind magnitude), the domain-mean surface wind is removed before computing those surface fluxes.

2. Automatic detection of squall line angle: method description

Figure 2 bottom panels in the paper shows the autocorrelation of the precipitable water at convective radiative equilibrium, for the sub-optimal $U_{sfc} = 2.5$ m s^{-1}, near-optimal $U_{sfc} = 10$ m s^{-1}, and super-optimal $U_{sfc} = 17.5$ m s^{-1} cases. In the sub-optimal case, we have the characteristic signal of a white noise, random, since we observe a peak in the center and a fast diffusion of the signal. In the optimal case, the orientation is along the y axis, consistent with the PW spatial distribution (top panels). In the super-critical case, a similar pattern is observed, with the two axes oriented at an angle. Thus the autocorrelation fields yield a centered representation of the convective organization. This next step will allow an automatic post-processing to measure the angle of the squall lines.

The angle a is defined as the angle between the squall line and the y-axis. To compute it, at each time step, we calculate the product between the spatial autocorrelation of PW (bottom contour plots of figure 2 in the main text) and a long (lengths 1 and 0.1 for major and minor axes) rotating 2D Gaussian oriented by a varying angle a. The selected angle a is the one that maximizes this product, i.e. that accounts for the best correlation.

By applying this method to all snaphots, we obtain a time distribution of the angle of the squall line with respect to the y-axis (between -$\pi/2$ and $\pi/2$). The mean of this distribution of angles in absolute value (between 0 and $\pi/2$) is our final angle, and its variance the incertitude (bottom curve in figure 2 of the main text).

Figure S1 shows the time evolution of the angle distribution for the three aforementioned cases. On the ordinate, we have the angle which varies from $-\pi/2$ to $\pi/2$, and on the abscissa, the time from the RCE equilibrium to the end of the simulation (5 days). Each pixel represents the probability density of an angle at a given time. In white, the evolution in time of the most probable angle is plotted. In the control case, we notice that the distributions are very wide, and decorrelated from one instant of time to another. In the sub-critical case, we have peaks around 0 during the whole evolution. In the super-critical case, we have three periods, first peaks of probability at -50, then a transition phase, and finally peaks in +50. This accounts for the indistinguishability between the 'right of the pool' and the 'left of the pool' for the projection of the angle, which sometimes gives rise to a broken line, similar to a V-shape.

From the time evolution of the angle distributions for each simulation, we can determine an averaged angle. We first reduced the angle to values between $[0, \pi/2]$ by taking its

absolute value. Therefore, the final angle selected by our automatic method is the average over time of the absolute value of the most probable angle. We associate to each angle a measurement uncertainty, which corresponds to the variance of the distribution of the most probable angle (again in absolute value) in time. This method yields the angle estimate versus shear strength shown figure 2 bottom panel of the main paper. We note that this method has been tested on several data sets and compared to repeated and rigorous manual measurements, which attests to the reliability of this approach.

3. Investigation of cold pool intensification with increasing background shear

Here we investigate whether the cold pool intensification with shear strength is due to the increase in depth, or to the increase in the buoyancy anomaly. We therefore calculate the contribution to changes in cold pool potential energy E_p from the height of the cold pool h and from the mean buoyancy anomaly in the cold pool E_p/h. To do this, we develop the energy as follows:

$$\frac{\Delta E_p}{E_p} = \frac{\Delta(E_p/h)}{(E_p/h)} + \frac{\Delta h}{h} + \mathcal{O}(\Delta^2) \tag{1}$$

where Δ is defined as

$$\Delta\phi = \frac{\phi_{ctrl} - \phi}{\phi_{ctrl}} \tag{2}$$

and Δ^2 denotes a second order error term (involving $\Delta(E_p/h) \times \Delta(h)$).

Figure S2 shows for each case this decomposition of the potential energy change. For example, for the case U_{sfc} = 20 m s^{-1}, we see that the potential energy has doubled compared to the control case; it has increased by 20% compared to the optimal case.

This figure shows that the evolution of the potential energy is mainly due to the increase of the height, with a smaller (but not negligible) contribution from increased buoyancy anomaly. We interpret these changes as resulting from the drier conditions, yielding higher and larger rain evaporation with increasing shear, as further discussed in the main text.

4. What are the origins of their intensification?

4.1. Investigation in rain evaporation profiles

The partial evaporation of rain, with its concomitant latent cooling is responsible for cold pool formation. We investigate rain evaporation profiles in the graph S3 where the vertical profiles of the relative humidity in dashed line, as well as the rain evaporation rate (QPEVP) in solid lines are displayed for each cases. The relative humidity field is calculated following Clausius-Clapeyron approximation. The rain evaporation rate is an output of the cloud resolving model SAM. We compute both these quantities in a composite way, i.e. we average in the vicinity of maximum precipitation at each time step. The vertical profiles are obtained by averaging in x and y near the maximum of precipitation (10km in x, 5km in y). In this graph, the thresholds QPEVP=34 g/kg/day and RH=0.9 are highlighted too.

We observe for highest shear case a maximum within the cold pool of $0.950 g/kg/day$ versus $0.830 g/kg/day$ for the control case. This intensification provokes both an increase in cold pool buoyancy anomaly and in the cold pool height. However, the latter is found to dominate (see Figure S2). The latent cooling is more intense and so downdrafts reach the isoline $b = -0.02K$ (cold pools upper boundary) higher in altitude.

In the graph S3 we can compare rain evaporation profiles and relative humidity, and observe that whereas rain evaporation increases with shear, the relative humidity decreases. This supports a causal link between these two quantity. In fact, dry conditions favors evaporation; and in the next subsection we propose a proper scaling of the rain evaporation in order to explain its intensification with shear.

4.2. Scaling of rain evaporation

We use the scaling of rain evaporation as the product of the distance to saturation and the ventilation function (following the default microphysics of SAM **??**).

$$QPEVP = \frac{\partial q_m}{\partial t}_{evap} = f(v_T)(1 - RH) \tag{3}$$

where: QPEVP is the rain evaporation, the rate of evaporation of precipitating water of type m (q_m); t is the time, $f(v_T)$ is the so called ventilation function which increases with v_T the terminal velocity, and RH is the relative humidity (RH).

We thus compute the evolution of the differential of the rain evaporation as a function of the shear strength. The differential is defined as $\Delta QPEVP = (QPEVP - QPEVP_{ctrl})/QPEVP_{ctrl}$. For each simulation,$\Delta QPEVP$ is decomposed into a contribution from relative humidity and ventilation function factor (and the second order error term), see Fig.S4. In this picture, we observe that the rain evaporation increases by 60% between the control case and the higher shear case. 40% of this increase is due to the drying of the air in this layer, and 20% corresponds to the ventilation function. This figure shows that the variation of rain evaporation is mainly the consequence of relative humidity decrease with shear.

The reasons why shear impacts relative humidity and the ventilation function deserves more investigation. We hypothesize that the relative humidity increase is due to changes in the meso-scale circulation which affects the relative humidity profiles. For the increase of ventilation factor, according to (?, ?), Appendix, Equation (A24),we can relate it to the precipitating water (of type rain, snow or graupel). In our case, the temperatures are warm enough at those heights for rain to dominate. Thus, $f(v_T)$ can be written as

$$f(v_T) \sim A_{em} q_r^{1/2} + B_{em} q_r^{5+bm/8}, \tag{4}$$

where q_r is the precipitating rain mixing ratio. We observe that q_r increases with the shear (see Figure S5), consistent with the increase of $f(v_t)$. The reason why the precipitating water increase with shear remains unclear and further work is needed.

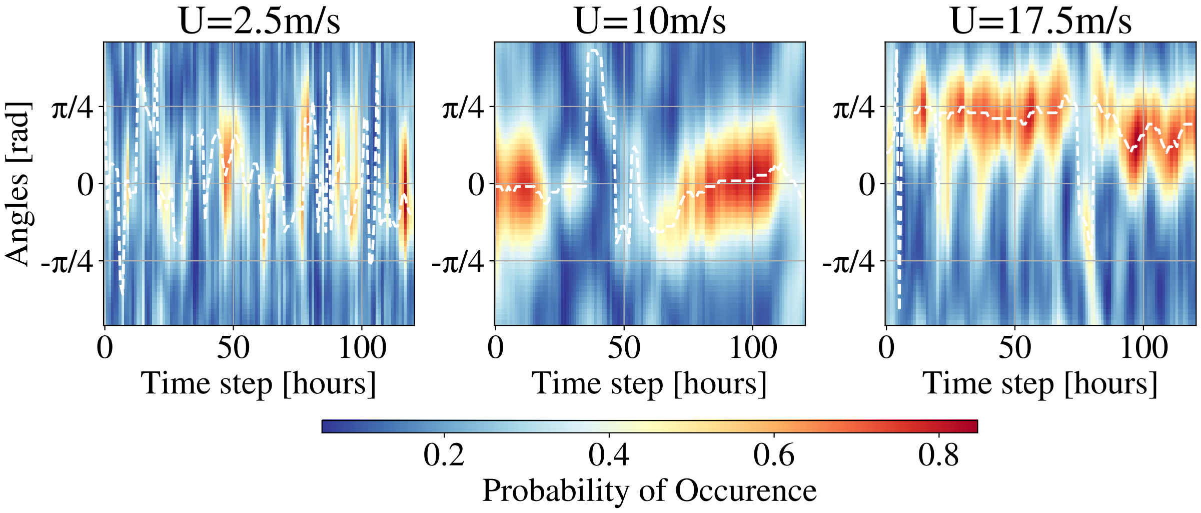

Figure S1. Time evolution of the distribution of angles of the squall lines with respect to the y-axis (perpendicular to the incoming wind) determined from the PW autocorrelation images.

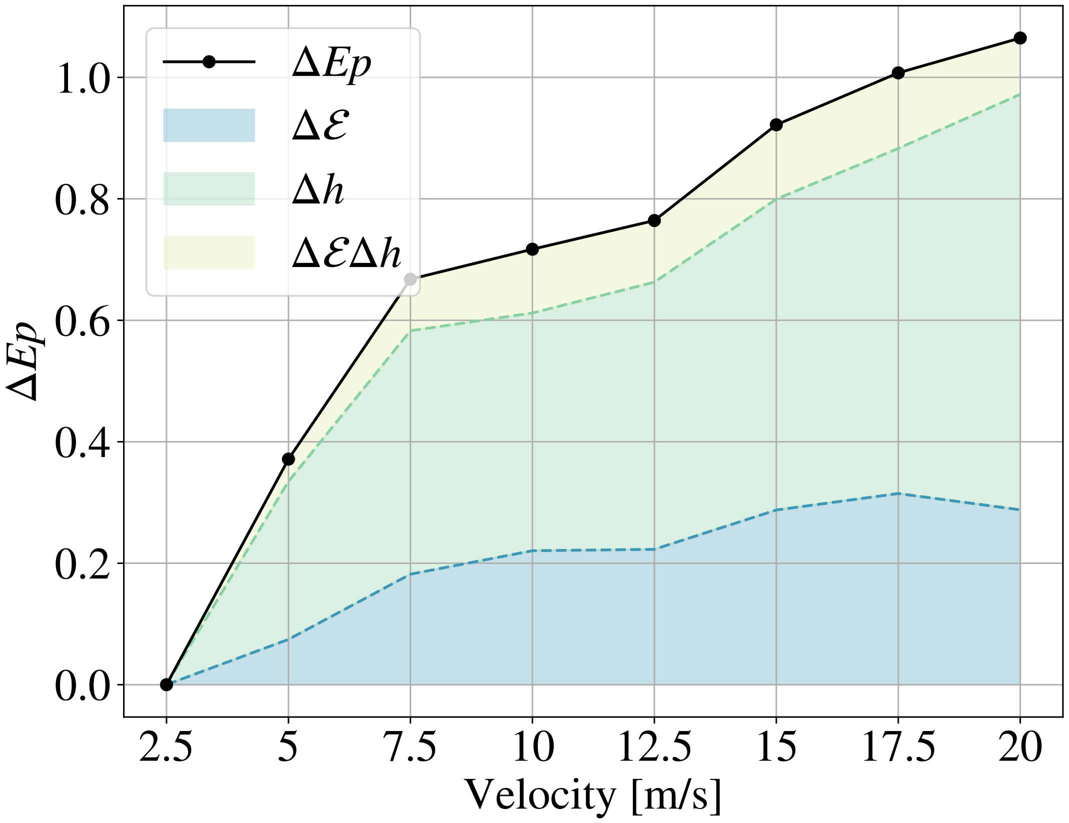

Figure S2. Evolution of the differential of the potential energy as a function of the shear strength. The differential is defined as $\Delta Ep = (Ep - Ep_{ctrl})/Ep_{ctrl}$. For each simulation, ΔEp is decomposed into a contribution from cold pool height h and cold pool strength $\mathcal{E} = E_p/h$ (and the second order error term). This figure shows that the intensification of the cold pools is mainly the consequence of deeper cold pools with increasing shear.

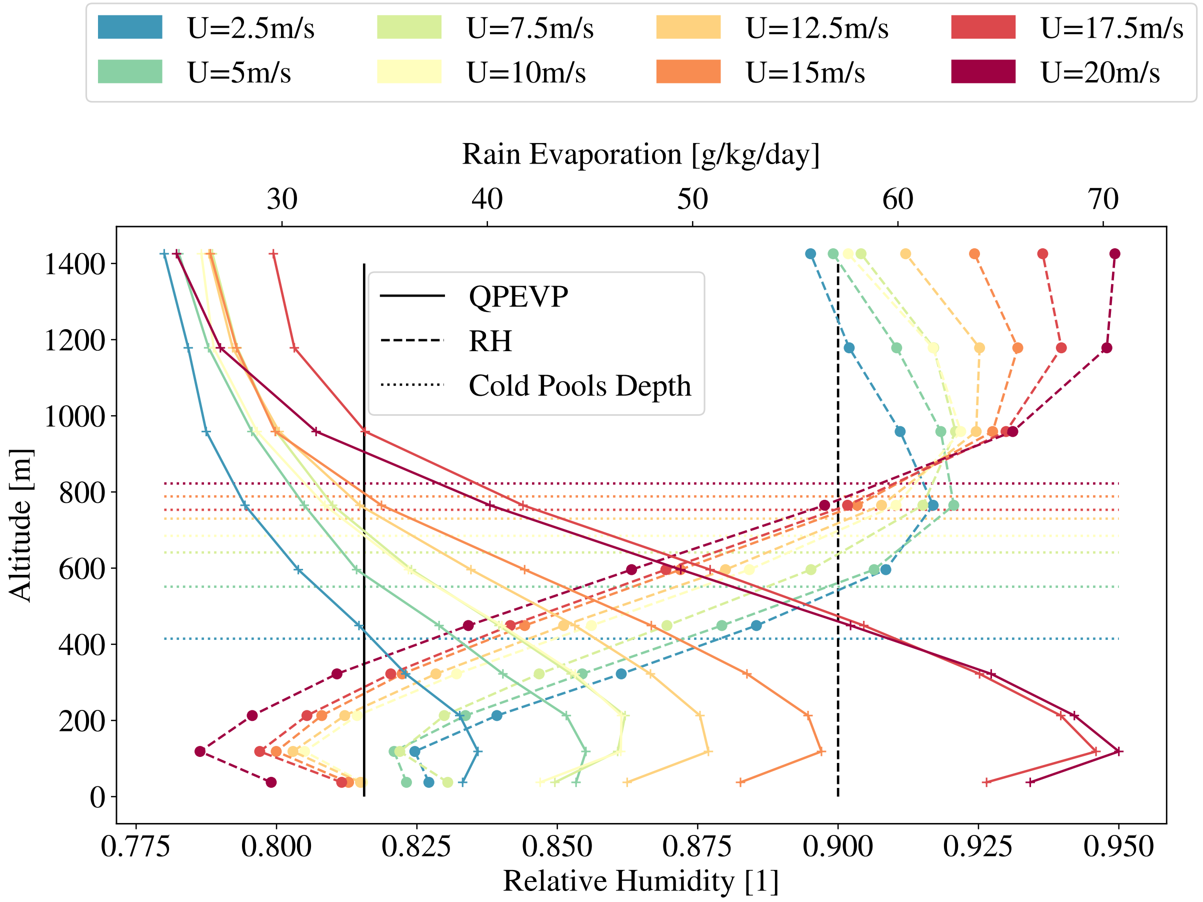

Figure S3. Vertical profiles of the relative humidity (dashed lines) and rain evaporation rate (solid lines). Cold pools depths are displayed in dotted lines. Each color represents a shear case. The threshold QPEVP=34g/kg/day and RH=0.9 are highlighted.

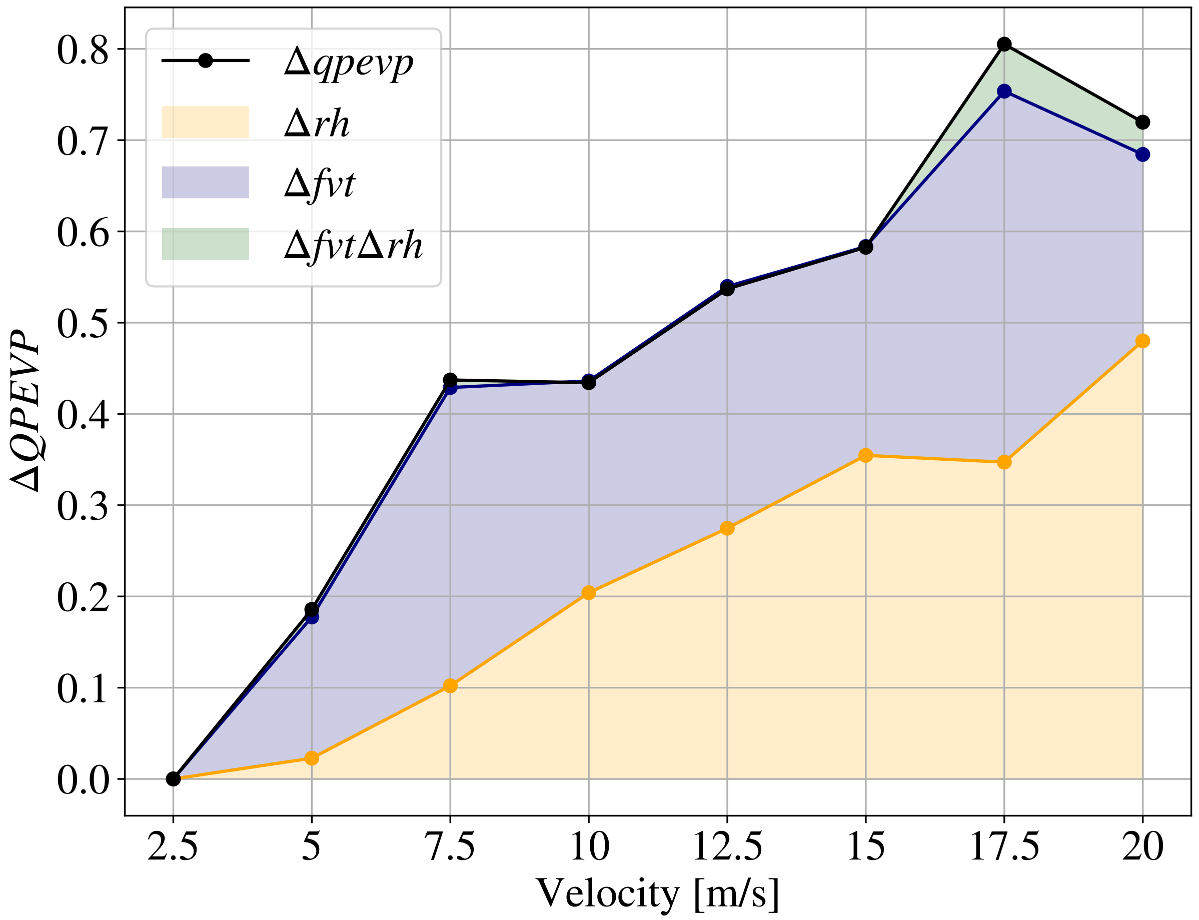

Figure S4. Evolution of the differential of the rain evaporation (QPEVP) as a function of the shear strength. The differential is defined as $\Delta A = (A - A_{ctrl}/A_{ctrl})$. For each simulation $\Delta QPEVP$ is decomposed into a contribution from the distance to saturation $(1 - RH)$ shortened to Δrh, and ventilation function $\Delta f(v_T)$, (and second order terms). This figure shows that intensification of rain evaporation is mainly due to the drying up with increasing shear.

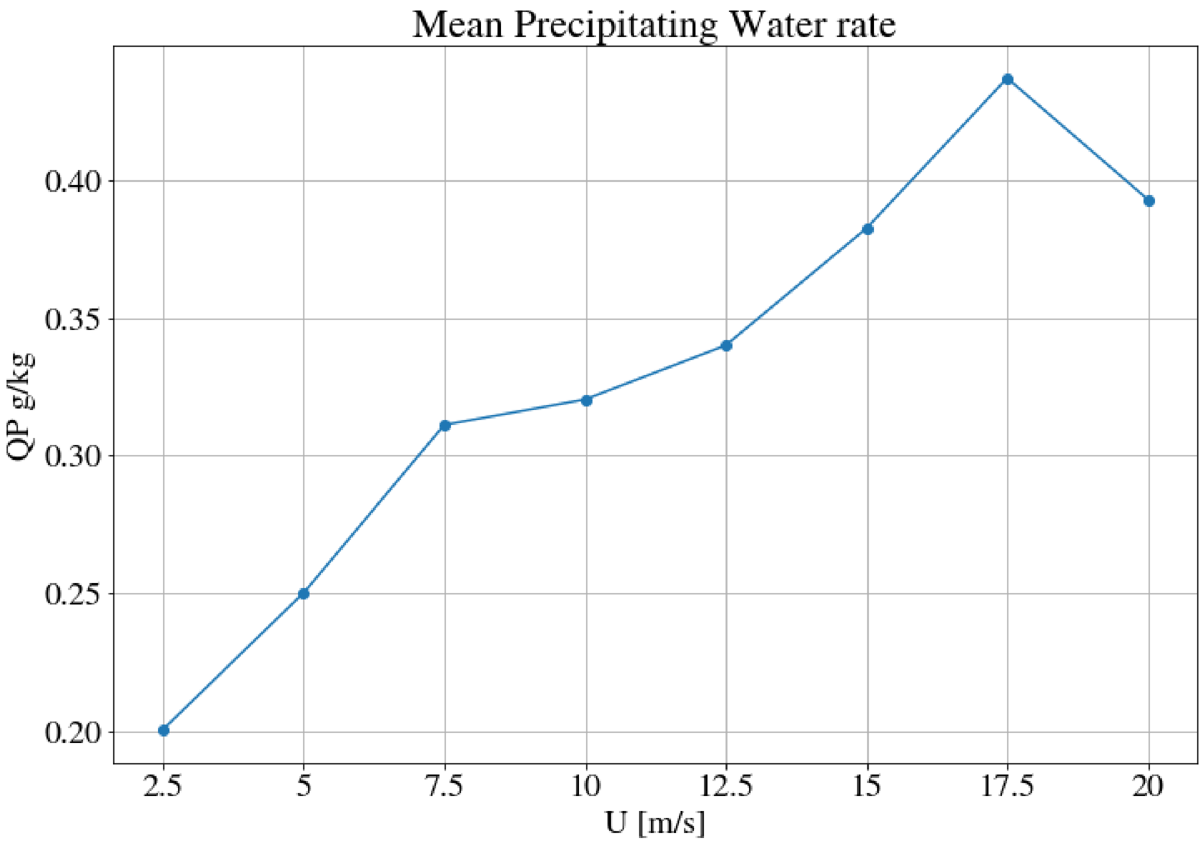

Figure S5. Evolution of the mean precipitating rain (QP) above cold pool as the shear increases. We observe that the stronger the shear, the more intense mean precipitating rain is. As there is more rain, provided the environment is dryer enough, rain evaporation is more intense and cold pool as well.
